# Dietary Sweet Sorghum (*Sorghum bicolor* (L.) Moench) Inclusion in Geese: Impacts on Growth Performance, Blood Biochemistry, and Intestinal Health

**DOI:** 10.3390/ani15121706

**Published:** 2025-06-09

**Authors:** Zuolan Liu, Xiaofeng Huang, Ying Chen, Jiajia Xue, Qun Xie, Hang Zhong, Yi Luo, Qigui Wang, Chao Wang

**Affiliations:** 1 Poultry Science Institute, Chongqing Academy of Animal Sciences, Rongchang, Chongqing 402460, China; liuzuolan9229@163.com (Z.L.); hxf2007@yeah.net (X.H.); chenying.cq@163.com (Y.C.); xuejiajia25@163.com (J.X.); xiequn20221208@163.com (Q.X.); hangzhongcqaas@163.com (H.Z.); homleestar@163.com (Y.L.); wangqigui@hotmail.com (Q.W.); 2 Scientific Observation and Experiment Station of Livestock Equipment Engineering in Southwest, Ministry of Agriculture, Rongchang, Chongqing 402460, China

**Keywords:** sweet sorghum, goose, growth performance, intestinal morphology, antioxidant capacity

## Abstract

This study investigated the effects of dietary sweet sorghum (SW) inclusion (0%, 4%, 8%, or 12%) on the growth performance, plasma biochemistry, antioxidant capacity, intestinal morphology, and duodenal digestive enzyme activity of geese. A total of 144 male geese (28 days old) were randomly divided into four groups (36 birds/group; six replicates). Experimental diets were formulated to contain 0%, 4%, 8%, or 12% SW to replace corn. The geese’s body weight and feed intake were recorded at 49 and 70 days, with samples collected at 70 days. The results showed that as SW levels increased, the geese’s average daily gain decreased during days 28–49 (*p* < 0.05), while their average daily feed intake (ADFI) and feed/gain ratio (F/G) increased during days 28–70 (*p* < 0.05). The cost of feed decreased with increasing SW levels, but the 12% SW group exhibited a higher feed cost/kg gain than the other groups (*p* < 0.05). The plasma biochemical parameters, antioxidant capacity, and duodenal digestive enzyme activity did not differ among the groups (*p* > 0.05). Geese fed 12% SW had higher duodenal villus heights than those in the 0% group (*p* < 0.05), and the jejunal muscularis thickness peaked in the 4% group (*p* < 0.05). The ileal morphology was unaffected (*p* > 0.05). SW increased the ADFI and F/G but had no adverse effects on plasma biochemistry, antioxidant status, or enzyme activity. Additionally, it improved duodenal and jejunal morphology. Based on the observed growth performance, feed cost/kg gain, and intestinal morphology, 8% dietary inclusion of SW is recommended.

## Simple Summary

Sweet sorghum is a high-quality forage crop with multiple benefits, including strong tolerance to stress, photosynthetic efficiency, a large biomass, richness in nutrients, and good palatability. Sweet sorghum has very good prospects for development in China. Sweet sorghum increased the ADFI and F/G in geese from 28 to 70 days of age and improved their duodenal and jejunal morphology. Sweet sorghum has no negative effect on plasma biochemical parameters, antioxidant capacity, or duodenal digestive enzyme activity in geese.

## 1. Introduction

China is the largest producer of geese in the world, with 515 million geese produced and a meat output value of CNY 53 billion in 2023 [1]. Therefore, the development of goose breeding is crucial to the development of animal husbandry in China. The rapid development of goose farming has been accompanied by an increasing shortage of poultry feed supply, leading to the high cost of traditional feed. The development and utilization of unconventional feed resources for geese are important to alleviate this problem and reduce the cost of feed [2].

As herbivorous waterfowl, geese efficiently utilize green forage due to their large, powerful gizzards and the microbial breakdown of fiber in their cecum and large intestine, which enables them to effectively digest high-fiber forage [3,4,5]. Additionally, a low-fiber diet has negative effects on growth performance, slaughter performance, serum biochemical parameters, and nutrient utilization in geese [6]. Sweet sorghum (SW, *Sorghum bicolor* (L.) Moench) is a high-quality forage crop with multiple benefits, such as stress tolerance, high photosynthetic efficiency, a large biomass, nutrient richness, and palatability [7,8]. SW has been widely used as a coarse feed in cattle [9,10,11], sheep [12,13,14], donkey [15], rabbit [16,17], and other animals [3], and the planting area has been expanded yearly in many places in China, providing very good prospects for development. Studies have confirmed that moderate SW supplementation improves growth performance [11,17], gastrointestinal tract development [16], nutrient digestibility [15], meat quality [11,17], and microbial diversity [4,16]. The main utilization methods of SW for ruminants include its incorporation into green feed, hay, and silage, but applications for geese are rare. Huang et al. [3] reported that geese fed a restricted commercial diet and ad libitum green SW stalks displayed changes in their growth performance, carcass and meat quality, and production economics. This feeding method, with geese picking green SW stalks to eat, results in a higher labor intensity due to the weighting of the commercial diet and cutting of fresh green SW stalks required every day.

Prior research has revealed that ryegrass or alfalfa meal pellet feed improves growth performance, carcass yield, and blood biochemical indices in geese [18,19,20]. However, few studies have investigated SW pellet feed in geese. Pelleting is the most common hydrothermal processing method used in the manufacturing of poultry diets, with its benefits including reduced feed wastage and nutrient segregation and increased feed intake, BW gain, bird uniformity, and feed efficiency [21,22,23]. Therefore, this study investigated the effects of pelleted feed containing SW on growth performance, plasma biochemistry, antioxidant capacity, and intestinal development in geese (aged 28–70 days).

## 2. Materials and Methods

### 2.1. Experimental Design and Bird Husbandry

All animal care and experimental procedures in this study were performed in accordance with the Regulations for the Administration of Affairs Concerning Experimental Animals of the People’s Republic of China and approved by the Animal Care and Welfare Committee of the Chongqing Academy of Animal Science (CAAS), China (approval number XKY-20220112). All geese used in this study were obtained from the CAAS goose-breeding center.

The SW cultivation area was in Rongchang district, China (29°15′~29°41′ N, 105°17′~105°44′ E; 380 m above sea level). The site has a subtropical climate with humid characteristics and good soil condition. Whole green SW at the early heading stage, 1.5 m in height, was mowed, chopped to 1–2 cm in size, and dried in the sun. The chemical composition of the dried SW is shown in Table 1. One-day-old male White Yuzhou goslings were reared in battery cage pens with a pen area of 0.65 m × 0.62 m at a stocking density of 6 goslings/pen. The ambient temperature was kept at 31 °C from 1 to 3 d of age and then decreased by 1 °C every 2 d until a temperature of 26 °C was reached. The lighting was continuous from 1 to 7 days of age. Then, it was reduced gradually to 16 h of light and 8 h of darkness. At the age of 15 days, the goslings were transferred to wire-floored pens with a pen size of 1.5 m^2^ (1 m × 1.5 m) at a stocking density of 9 goslings/pen. The lighting program remained unchanged. The ambient temperature ranged from 20 °C to 25 °C, and the humidity ranged from 65% to 81%. The goslings were fed commercial corn-soybean-based diets formulated according to breed requirements with 19% crude protein, 11.7 MJ/kg metabolizable energy, and 1% calcium from 1 to 28 days of age. Water was provided using a drip nipple water supply line, and pelleted feed was provided. The goslings had free access to water and feed. A total of 144 male White Yuzhou geese (28 days old) were randomly assigned to four dietary treatments containing SW, with six replicate pens per treatment and six geese per pen. The experimental diets were formulated to include 0%, 4%, 8%, or 12% dried SW to replace corn.

The experimental diets were formulated mainly according to the NRC [24] recommendations and prior research results regarding major nutrients for geese [25]. The compositions and nutrient levels of the experimental diets are listed in Table 2. The diet was provided in a pelleted form according to the feed formula via crushing, mixing, and granulation. Water was supplied through nipple drinkers. The geese were reared in the same houses with plastic wire floors and had free access to water and feed during the whole experimental period. The lighting program was 16 h of light and 8 h of darkness. The ambient temperature and humidity were monitored using a temperature and humidity recorder at 4 h intervals. The ambient temperature ranged from 18 °C to 30 °C, and the humidity ranged from 65% to 81%. Standard management procedures were used throughout the experiment.

### 2.2. Sample Collection and Analytical Determination

#### 2.2.1. Growth Performance

The growth performance of the geese was evaluated based on their body weight (BW), average daily feed intake (ADFI), average daily gain (ADG), and feed/gain ratio (F/G). The BW was measured after a 12-hour fasting period (with water available) using an electronic scale (YH-T1, Yingheng, Huizhou, China; capacity: 70 kg, accuracy: 1 g) at 28, 49, and 70 days of age. The feed intake was recorded per pen, and the ADFI and F/G were adjusted for mortality. The ADG, ADFI, and F/G were calculated for the periods 28–49 d, 49–70 d, and 28–70 d.

#### 2.2.2. Plasma Biochemical Parameters

At 70 days of age, blood samples were collected from 6 geese with a weight close to the average weight of the treatment (*n* = 6; each treatment was represented by 6 replicates, with 1 bird per replicate) after a 12-hour fasting period. Approximately 5 mL of blood was drawn from the jugular vein using anticoagulant vacuum tubes. Plasma was separated by means of centrifugation (3500 rpm, 15 min, 4 °C; 5424R, Eppendorf, Shanghai, China) and stored at −80 °C for biochemical analysis. Alanine amino transferase (ALT), aspartate amino transferase (AST), alkaline phosphatase (ALP), lactate dehydrogenase (LDH), blood urea nitrogen (BUN), uric acid (UA), cholesterol (CHOL), triglyceride (TG), high-density lipoprotein (HDL), low-density lipoprotein (LDL), and glucose (GLU) in the plasma were determined with the colorimetric method using an automatic biochemical analyzer (AU680, Beckman Coulter, Tokyo, Japan) with corresponding commercial kits (ALT, C009-3-2; AST, C010-3-2; ALP, A059-3-1; LDH, A020-5-2; BUN, C013-2-1; UA, C012-2-1; CHOL, A111-1-1; TG, A110-1-1; HDL, A112-1-1; LDL, C113-1-1; GLU, A154-1-1; Nanjing Jiancheng Bioengineering Institute, Nanjing, China) following the manufacturer’s guides. The activities of ALT, AST, ALP, and LDH were measured using the continuous monitoring method. The concentrations of BUN, UA, CHOL, TG, HDL, LDL, and GLU were measured using the endpoint method.

#### 2.2.3. Antioxidant Capacity

At 70 days of age, one goose per pen (with a body weight close to the pen’s average) was selected for euthanasia by means of cervical dislocation. Before sampling, the geese were deprived of feed for 12 h but had access to water. Blood and liver samples were collected to assess antioxidant status. The total antioxidant capacity (T-AOC), content of malondialdehyde (MDA), and activity of glutathione peroxidase (GSH-Px), superoxide dismutase (SOD), and catalase (CAT) in the plasma and liver were detected using commercial analytical kits according to the manufacturer’s recommendations (T-AOC, A015-2-1; MDA, A003-1-1; GSH-Px, A005-1-2; SOD, A001-1-1; CAT, A007-1-1; Nanjing Jiancheng Bioengineering Institute, Nanjing, China).

#### 2.2.4. Intestinal Morphology

Intestinal segments were excised from the slaughtered geese. Duodenum, jejunum, and ileum segments were collected and analyzed according to the methods used in our previous studies [26]. Briefly, 1 cm sections from the middle portion of the duodenum, jejunum, and ileum tissues were fixed in 10% formaldehyde phosphate buffer after washing with 0.1 M phosphate-buffered saline. The fixed sections were processed, dehydrated, and embedded in paraffin wax. Then, the samples were sectioned at 5 μm and stained with hematoxylin-eosin. Histological sections were examined for their villus height (VH), crypt depth (CD), and muscularis thickness (MT), which were determined on 10 well-oriented villi and 10 muscularis thicknesses chosen from each segment, using a digital camera microscope (BA400 Digital, McAudi Industrial Group Co., Ltd., Xiamen, China) and the Motic Advanced 3.2 digital image analysis system. The ratio of the villus height to the crypt depth (VH/CD) was subsequently calculated.

#### 2.2.5. Digestive Enzyme Activity

The duodenal mucosa was gently scraped using a sterile glass slide, collected in 1.5 mL sterile tubes, immediately frozen in liquid nitrogen, and stored at −80 °C until analysis. The activities of cellulase, chymotrypsin, amylase, lipase, and trypsin were measured using commercial assay kits (Nanjing Jiancheng Bioengineering Institute, Nanjing, China; catalog numbers: cellulase, A138-1-1; chymotrypsin, A080-3-1; amylase, C016-1-2; lipase, A054-2-1; trypsin, A080-2-2) following the manufacturer’s protocols.

### 2.3. Statistical Analysis

The data were analyzed by applying the one-way ANOVA procedure in SPSS 21.0 software (SPSS Inc., Chicago, IL, USA). The growth performance was analyzed with the pen used as the experimental unit. Plasma biochemical parameters, antioxidant capacity, intestinal morphology, and duodenal digestive enzyme activity were analyzed on an individual basis. When the SW treatment was significant, the means were compared using Duncan’s multiple range test. In addition, orthogonal polynomial contrasts were used to assess the significance of linear or quadratic models to describe the response of the dependent variable to dietary SW levels. All statements of differences are based on a significance level of *p* < 0.05.

## 3. Results

### 3.1. Growth Performance

The effects of the SW level on the growth performance of the geese are presented in Table 3. From 28 to 49 days of age, no significant differences were observed in BW (*p* > 0.05), except for a trend (*p* = 0.036) toward lower BW with increasing SW levels. The geese fed the diet containing 12% SW exhibited a lower ADG and higher F/G than the other groups (*p* < 0.05). From 49 to 70 days of age, the geese fed the diet containing 12% SW showed a higher ADFI compared with the other groups (*p* < 0.05). From 28 to 70 days of age, the geese fed the diet containing 12% SW had a higher ADFI and F/G than the other groups (*p* < 0.05). The F/G increased linearly or quadratically with increasing SW levels (*p* < 0.05), but no significant difference was observed between geese fed diets with 0% and 4% SW. The cost of feed decreased with increasing SW levels, but the 12% SW group exhibited a higher feed cost/kg gain than the other groups (*p* < 0.05). This suggests that the optimal dietary SW level is 8%.

### 3.2. Plasma Biochemical Parameters and Antioxidant Capacity

The effects of the SW level on the geese’s plasma biochemical parameters are presented in Table 4. The dietary SW level did not affect the plasma ALT, AST, ALP, and LDH activities or plasma BUN, UA, CHOL, TG, HDL, LDL, and GLU concentrations in the geese from 28 to 70 d of age (*p* > 0.05). The effects of SW level on the antioxidant capacity of the geese are presented in Table 5. No significant differences were observed in T-AOC, MDA, SOD, GSH-Px (*p* = 0.093), or CAT (*p* = 0.064) in the plasma or liver (*p* > 0.05), except for a trend toward higher GSH-Px and CAT activity in plasma with increasing SW levels.

### 3.3. Intestinal Morphology and Duodenal Digestive Enzyme Activity

The effects of SW level on the intestinal morphology of the geese are presented in Table 6. The geese fed the diet containing 12% SW had higher duodenal VHs than those fed 0%. The jejunal MT presented the highest value in the 4% group (*p* < 0.05). No significant differences were observed in the CD, VH/CD, or MT of the duodenum or in the VH, CD, or VH/CD of the jejunum (*p* > 0.05). The SW had no effect on ileal morphology (*p* > 0.05). The effects of SW level on the duodenal digestive enzyme activity of the geese are presented in Table 7. There were no differences in the activity of cellulase, trypsin, chymotrypsin, lipase, or amylase in the duodenum among geese fed SW at levels of 0%, 4%, 8%, and 12% (*p* > 0.05).

## 4. Discussion

### 4.1. Growth Performance

Due to its high yield and strong ability to adapt to drier environments, many scholars are exploring SW as a forage type in ruminant production; however, research related to its utilization in poultry production is limited. In dairy cows, Yu [10] and Lv et al. [27] observed increased dry matter intake with higher proportions of SW silage, a trend consistent with our findings in geese, where the ADFI rose as dietary SW levels increased. This increase in the ADFI suggests that SW is more palatable and can increase the growth of the gastrointestinal tract in geese to increase space for further intake [3]. In contrast, Li et al. [28] and Ran et al. [29] reported a linear decrease in dry matter intake in dairy cows fed higher levels of SW silage. These discrepancies may stem from differences in silage processing methods or forage maturity at harvest. The impact of SW on feed intake and growth performance in animals has been found to vary. In fattening cattle, SW can improve weight gain and decrease the F/G, without any effect on feed intake [11]. In lambs, SW stover is a newer and cheaper untapped roughage source that could be utilized in complete rations processed into a mash form, without affecting growth performance [30]. Wang et al. [14] reported similar findings: SW silage did not alter the ADFI or ADG in sheep. However, Wu et al. [31] reported that lambs fed a SW silage diet exhibited decreased feed intake and weight gain, resulting in a significant increase in the F/G. SW with corn mixed silage can increase the ADFI and ADG and decrease the F/G in sheep [12]. These inconsistent results regarding SW’s effects on animals’ growth performance may be attributed to several factors, including the SW harvest date, diet formulation, processing style, and animal species. Consequently, further research on this topic is necessary to provide better references for practitioners.

The decreased BWs observed among the geese at 28 to 49 days of age were compensated at 49 to 70 days of age, such that the BW of geese in the 12% group was similar to that of geese in the other groups. This finding aligns with the results of a study conducted by Kokoszyński et al. [32], who reported that geese fed with restricted amounts of commercial diets and ad libitum amounts of maize silage displayed a decrease in BW at the end of the rearing period; however, the geese compensated for this effect during the oat fattening period. In our study, increasing the level of SW in the diet increased its fiber content. The geese fed the 12% SW diet exhibited lower ADGs from 28 to 49 days of age compared to the other groups. However, from 49 to 70 days, their growth rate matched those of the other groups, suggesting metabolic adaptation to the high-fiber diet. The lower BW and ADG of geese fed with the diet containing 12% SW in the first 3 weeks of rearing might have occurred because the digestive tract was not yet adapted to digesting the higher fiber content. Adaptation to an increased dietary fiber content increases with the age of the birds and with the development of the digestive tract [33]. The increased F/G in geese fed higher SW levels likely resulted from elevated ADFIs without significant improvements in ADGs. This is in accordance with results from Wang et al. [34], who reported that substituting roughage (whole-plant silage maize) for commercial feed significantly increased the ADFI and F/G of geese. It is essential to note that the cost of the feed decreased with increasing SW levels, while SW increased feed consumption; thus, the feed cost/kg gain in the 12% SW group was the highest. Therefore, SW may serve as a cost-effective alternative feed ingredient for geese, provided that its inclusion level does not exceed 8% to avoid adverse effects on the feed cost/kg gain.

### 4.2. Plasma Biochemical Parameters and Antioxidant Capacity

Blood metabolic by-products, enzyme activity, and antioxidant activity can be used to evaluate the metabolic function and health status of an animal [35]. AST, ALT, and ALP are often specifically expressed in the liver, heart, and muscle. Their enzyme activity in the blood remains stable at low levels under normal circumstances; an abnormal increase in this activity indicates organ damage [29]. LDH is widely present in the cytoplasm and can catalyze the oxidative decomposition of lactate to provide energy for the body [36]. The stable plasma ALT, AST, and ALP activities observed in our study (Table 4) indicate that the SW diets did not induce hepatic or muscular damage in the geese. This result is in line with results from Ran et al. [29], who reported that the substitution of corn silage with SW silage did not affect blood ALT or ALP activity in dairy cows. In addition, a higher plasma UA level was positively correlated with long-term purine metabolism disorder, which eventually led to gout [37]. BUN is a product of protein metabolism, and its level can increase due to a high catabolic state [38]. The indicators of lipid metabolism are CHOL, TG, HDL, and LDL; GLU is an indicator of glucose metabolism. Our results also showed that plasma TG, CHOL, HDL, LDL, BUN, UA, and GLU concentrations were not affected in geese fed SW, which indicates that SW has no adverse effect on protein, lipid, or glucose metabolism in geese. This result is basically consistent with previous findings relating to dairy cows [9] and Dezhou donkey foals [15].

T-AOC, GSH-Px, SOD, and CAT are all considered vital indicators of antioxidant status in the body; they can prevent oxidative stress by eliminating free radicals such as reactive oxygen species [39]. MDA has been recognized as a marker of oxidative stress and the final product of lipid peroxidation, which indirectly reflects the degree of cellular damage [40,41]. Oxidative stress can inhibit the growth of animals and lead to production loss. In the present study, no difference was observed in the plasma antioxidant capacity of geese fed different levels of SW in their diets. Hence, this conforms to the result that SW did not change the BW or ADG of the geese from 28 to 79 d of age, and SW can be used as a forage source for geese. In line with this observation, providing an SW silage diet did not affect the total antioxidant capacity of blood in dairy cows [29]. However, a study by Yu [10] reported that SW silage decreased serum T-AOC and increased the MDA concentration, resulting in a decrease in antioxidant capacity in dairy cows. The SW silage diet has been reported to improve the antioxidant capacity of blood in Holstein dairy cows [42] and mutton sheep [14]. The discrepancies between these results and those of our current study might be attributed to differences in the SW harvest dates and processing methods, as well as the use of animals of different ages or genetic backgrounds. For instance, Khosravi et al. [42] reported improved antioxidant capacity in cows fed sorghum silage harvested at early maturity stages, whereas our study used sun-dried SW harvested at the heading stage, which may explain the divergent outcomes.

### 4.3. Intestinal Morphology and Duodenal Digestive Enzyme Activity

The small intestine is the main site of nutrient digestion and absorption in geese, and small intestinal morphology is related to the digestion and absorption of different substances [38,43]. An increase in VH increases the contact area between the small intestine and nutrients, which facilitates digestion and absorption. A shallow CD means a decrease in absorption functions. An increase in VH/CD indicates an improvement in the intestinal mucosal structure, promoting digestion and absorption, and a larger MT is an indicator of better nutrient absorption [35,44]. In the present study, SW increased the duodenal VH. The diet supplemented with 4% SW increased the jejunal MT, but increasing the level to 12% decreased the jejunal MT again. These results are basically similar to findings reported by Chen [16], who noted that a content of 20% mixed fermented feed (a 75:25 ratio of machine-picked cotton residue to SW) can promote small intestine morphological development in Ira meat rabbits. The increased VH in the duodenum can increase the surface area available for the absorption of nutrients [45], which is consistent with the compensated BW and greater ADFI of the geese. Further investigations are necessary to clarify the effects and the underlying mechanisms through which SW regulates the jejunal MT of geese.

The digestive enzymes in the gut have key functions in the degradation of nutrients [38]. Our results showed that SW did not influence digestive enzyme activities in the duodenum in geese, indicating that the utilization of protein, starch, and fat was not affected by dietary SW supplementation. In support of this, the plasma BUN, UA, GLU, CHOL, HDL, LDL, and TG concentrations in the geese showed no differences among the treatments. In the present study, SW did not harm the digestion and absorption of nutrients. Therefore, SW is feasible and safe as a feed ingredient for geese. A recent study in Ira meat rabbits found that a content of 20% mixed fermented feed (a 75:25 ratio of machine-picked cotton residue to SW) increased digestive enzyme activity [16]. However, another study reported negative effects of SW on digestive enzyme activity. It found that with an increasing proportion of SW in mixed silage, digestive enzyme activity in the digestive tract of karakul sheep was significantly decreased [13]. These contradictions in results may be due to differences among breeds and SW processing methods.

## 5. Conclusions

Our results demonstrated that dietary SW increased feed intake and the F/G while showing no negative effects on the ADG over the total experimental period or on plasma biochemical parameters, antioxidant capacity, or duodenal digestive enzyme activity in geese. Additionally, it improved the duodenal and jejunal morphology. Based on the observed growth performance parameters and intestinal morphology improvements, we recommend an 8% dietary inclusion level of SW as the optimal concentration for goose nutrition.

## Figures and Tables

**Table 1:** Analyzed nutrient contents of sweet sorghum in this study.

Items, % of DM	Content	Items, % of DM	Content
Gross energy (MJ/kg)	17.8	Methionine	0.11
Crude protein	11.94	Threonine	0.46
Crude fat	2.57	Arginine	0.54
Crude fiber	43.75	Valine	0.60
Ash	8.06	Isoleucine	0.44
Calcium	0.42	Leucine	0.87
Total phosphorus	0.32	Phenylalanine	0.72
Lysine	0.65	Histidine	0.23

**Table 2:** Compositions and nutrient levels of experimental diets (air-dry basis).

Items	Sweet Sorghum Level			
	0%	4%	8%	12%
Ingredients, %				
Corn	60.00	56.00	52.00	48.00
Sweet sorghum (air-dry basis)	0.00	4.00	8.00	12.00
Soybean meal	17.00	16.70	16.40	16.10
Wheat bran	19.30	19.35	19.40	19.45
Soybean oil	0.00	0.30	0.60	0.90
Limestone	1.10	1.05	1.00	1.00
Calcium hydrogen phosphate	1.40	1.40	1.40	1.35
Sodium chloride	0.30	0.30	0.30	0.30
*L*-Lysine hydrochloride	0.10	0.10	0.10	0.10
*L*-Methionine	0.20	0.20	0.20	0.20
Tryptophan	0.10	0.10	0.10	0.10
Threonine	0.10	0.10	0.10	0.10
Choline chloride	0.10	0.10	0.10	0.10
Mineral and vitamin premix ^1^	0.30	0.30	0.30	0.30
Total	100	100	100	100
Measured nutrient levels, %				
Crude protein	16.02	15.98	15.93	15.88
Crude fiber	4.31	5.78	7.24	8.71
Calcium	0.81	0.81	0.80	0.80
Total phosphorus	0.66	0.66	0.66	0.66
Lysine	0.84	0.85	0.85	0.86
Methionine	0.46	0.45	0.45	0.44
Threonine	0.69	0.69	0.69	0.69
Calculated nutrient levels, %				
Metabolizable energy (MJ/kg)	10.96	10.96	10.96	10.96
Tryptophan	0.30	0.29	0.29	0.28
Nonphytate phosphorus	0.35	0.35	0.35	0.33

^1^ The premix provided the following per kg of diet: Cu (CuSO_4_ 5H_2_O) 8 mg; Fe (FeSO_4_ H_2_O) 96 mg; Zn (ZnSO_4_ H_2_O) 80 mg; Mn (MnSO_4_ H_2_O) 100 mg; Se (Na_2_SeO_3_) 0.3 mg; I (KI) 0.4 mg; vitamin A 6000 IU; vitamin D_3_ 1500 IU; vitamin E 10 IU; vitamin K_3_ 2.4 mg; vitamin B_1_ 1.5 mg; vitamin B_2_ 5 mg; vitamin B_6_ 3 mg; vitamin B_12_ 0.02 mg; pantothenic acid 10 mg; nicotinic acid 50 mg; folic acid 0.5 mg; biotin 0.15 mg.

**Table 3:** Effects of sweet sorghum level on the growth performance of geese from 28 to 70 days of age.

Item ^1^	Day	Sweet Sorghum Level				SEM	*p*-Value		
		0%	4%	8%	12%		*ANOVA*	*Linear*	*Quadratic*
BW (g/bird)	28	1110.9	1111.5	1111.9	1117.2	1.70	0.566	0.233	0.503
	49	2461.1	2501.5	2453.0	2377.8	16.7	0.054	0.036	0.066
	70	3065.4	3109.0	3048.1	3033.5	15.4	0.353	0.264	0.351
ADG (g/bird/day)	28–49	64 ^a^	65.6 ^a^	63.9 ^a^	59.4 ^b^	0.77	0.017	0.014	0.027
	49–70	28.8	28.9	28.3	31.2	0.52	0.197	0.148	0.175
	28–70	46.3	47.4	46.1	45.4	0.36	0.316	0.232	0.245
ADFI (g/bird/day)	28–49	171.7	175.4	177.4	178.8	1.40	0.316	0.073	0.684
	49–70	167.7 ^b^	176.3 ^b^	174.0 ^b^	187.1 ^a^	1.96	0.001	<0.001	0.436
	28–70	169.7 ^b^	175.8 ^b^	175.7 ^b^	182.9 ^a^	1.44	0.006	0.001	0.810
F/G (g/g)	28–49	2.69 ^b^	2.68 ^b^	2.78 ^b^	3.01 ^a^	0.03	<0.001	<0.001	0.002
	49–70	5.84	6.11	6.17	6.03	0.08	0.555	0.420	0.241
	28–70	3.67 ^c^	3.71 ^bc^	3.81 ^b^	4.03 ^a^	0.03	<0.001	<0.001	0.036
Cost of feed/1000 kg (USD)		463.4	455.3	447.2	438.9	/	/	/	/
Feed cost/kg gain (USD)		1.70 ^a^	1.69 ^a^	1.71 ^a^	1.77 ^b^	0.01	0.010	0.005	0.045

^a, b, c^ In the same row, values with different lowercase letter superscripts are significantly different (*p* < 0.05), while those with common or no letter superscripts are not significantly different (*p* > 0.05). ^1^ Data are means of six replicates, with six birds per replicate. Abbreviations: BW, body weight; ADG, average daily gain; ADFI, average daily feed intake; F/G, feed/gain ratio.

**Table 4:** Effects of sweet sorghum level on plasma biochemical parameters of geese at 70 days of age.

Item ^1^	Sweet Sorghum Level				SEM	*p*-Value		
	0%	4%	8%	12%		*ANOVA*	*Linear*	*Quadratic*
ALT (U/L)	9.50	12.33	10.83	11.50	0.63	0.463	0.437	0.403
AST (U/L)	24.2	22.3	26.0	23.8	1.04	0.693	0.784	0.939
ALP (U/L)	1366.2	1374.5	1431.8	1250.2	81.8	0.899	0.711	0.589
LDH (U/L)	402.3	390.5	400.0	414.3	19.2	0.982	0.807	0.753
BUN (mmol/L)	0.42	0.36	0.46	0.41	0.02	0.581	0.773	1.000
UA (μmol/L)	264.5	233.5	235.8	217.7	8.89	0.322	0.093	0.718
CHOL (mmol/L)	4.13	4.28	4.07	3.62	0.13	0.306	0.136	0.244
TG (mmol/L)	0.64	0.64	0.48	0.62	0.05	0.585	0.618	0.500
HDL (mmol/L)	2.24	2.16	2.30	2.00	0.06	0.360	0.299	0.392
LDL (mmol/L)	1.53	1.74	1.40	1.26	0.07	0.093	0.060	0.204
GLU (mmol/L)	9.94	9.38	10.05	8.60	0.26	0.171	0.139	0.371

^1^ Data are means of six replicates, with one bird per replicate. Abbreviations: ALT, alanine aminotransferase; AST, aspartate aminotransferase; ALP, alkaline phosphatase; LDH, lactate dehydrogenase; BUN, blood urea nitrogen; UA, uric acid; CHOL, cholesterol; TG, triglyceride; HDL, high-density lipoprotein; LDL, low-density lipoprotein; GLU, glucose.

**Table 5:** Effects of sweet sorghum level on the antioxidant capacity of geese at 70 days of age.

Item ^1^	Sweet Sorghum Level				SEM	*p*-Value		
	0%	4%	8%	12%		*ANOVA*	*Linear*	*Quadratic*
Plasma								
T-AOC (mmol/mL)	1.22	1.25	1.23	1.23	0.01	0.856	0.869	0.652
GSH-Px (U/mL)	291.8	316.9	328.2	371.3	11.6	0.093	0.016	0.676
SOD (U/mL)	1187.5	1125.0	1173.7	1094.5	20.9	0.383	0.230	0.843
CAT (U/mL)	0.32	0.37	0.44	0.40	0.02	0.064	0.031	0.168
MDA (nmol/mL)	4.33	4.06	3.98	3.83	0.10	0.336	0.080	0.748
Liver								
Protein concentration (mgprot/mL)	10.13	9.41	9.58	9.80	0.19	0.608	0.643	0.247
T-AOC (mmol/gprot)	0.19	0.20	0.21	0.20	0.01	0.720	0.567	0.370
GSH-Px (U/mgprot)	208.8	201.9	230.3	216.7	5.08	0.235	0.248	0.739
SOD (U/mgprot)	722.6	801.5	785.4	761.9	18.3	0.479	0.546	0.182
CAT (U/mgprot)	37.2	39.5	39.6	36.7	0.68	0.312	0.791	0.069
MDA (nmol/mgprot)	0.59	0.69	0.61	0.66	0.03	0.638	0.646	0.631

^1^ Data are means of six replicates, with one bird per replicate. Abbreviations: T-AOC, total antioxidant capacity; GSH-Px, glutathione peroxidase; SOD, superoxide dismutase; CAT, catalase; MDA, malondialdehyde.

**Table 6:** Effects of sweet sorghum level on the intestinal morphology of geese at 70 days of age.

Item ^1^	Sweet Sorghum Level				SEM	*p*-Value		
	0%	4%	8%	12%		*ANOVA*	*Linear*	*Quadratic*
Duodenum								
VH (μm)	712.4 ^b^	768.4 ^ab^	799.2 ^ab^	918.8 ^a^	28.2	0.045	0.012	0.774
CD (μm)	137.6	148.2	135.6	155.6	4.42	0.356	0.302	0.599
MT (μm)	536.7	496.0	460.7	477.2	13	0.189	0.067	0.261
VH/CD	5.22	5.41	6.16	6.04	0.25	0.498	0.175	0.762
Jejunum								
VH (μm)	1011.4	1069.2	992.1	1193.1	34.6	0.159	0.125	0.286
CD (μm)	111.6	116.2	118.3	117.6	2.93	0.868	0.476	0.669
MT (μm)	344.1 ^b^	498.9 ^a^	405.2 ^ab^	370.9 ^b^	21.4	0.045	0.938	0.021
VH/CD	9.08	9.68	8.59	10.24	0.45	0.617	0.565	0.574
Ileum								
VH (μm)	653.5	631.4	736.3	750.6	25.4	0.259	0.087	0.716
CD (μm)	126.7	125.8	127.6	132.9	3.40	0.898	0.533	0.674
MT (μm)	423.3	459.7	435.7	473.5	14.8	0.647	0.363	0.981
VH/CD	5.13	5.10	5.85	5.67	0.19	0.406	0.173	0.849

^a, b^ In the same row, values with different lowercase letter superscripts are significantly different (*p* < 0.05), while those with common or no letter superscripts are not significantly different (*p* > 0.05). ^1^ Data are means of six replicates, with one bird per replicate. Abbreviations: VH, villus height; CD, crypt depth; MT, muscularis thickness, VH/CD, villus-height- to-crypt-depth ratio.

**Table 7:** Effects of sweet sorghum level on duodenal digestive enzyme activity of geese at 70 days of age.

Item ^1^	Sweet Sorghum Level				SEM	*p*-Value		
	0%	4%	8%	12%		*ANOVA*	*Linear*	*Quadratic*
Protein concentration (mgprot/mL)	3.45	3.36	3.45	3.78	0.11	0.585	0.301	0.365
Cellulase (U/mgprot)	2.97	2.91	2.68	2.69	0.15	0.876	0.454	0.911
Trypsin (U/mgprot)	574.5	557.9	450.4	458.5	38.4	0.570	0.208	0.877
Chymotrypsin (U/mgprot)	0.78	0.91	0.88	0.80	0.07	0.891	0.941	0.451
Lipase (U/gprot)	4.95	5.23	5.79	5.12	0.24	0.659	0.639	0.350
Amylase (U/mgprot)	1.79	2.16	1.84	1.83	0.12	0.674	0.866	0.431

^1^ Data are means of six replicates, with one bird per replicate.

## Data Availability

All relevant data are within the manuscript.

## Abbreviations

The following abbreviations are used in this manuscript:

SW	Sweet sorghum
BW	Body weight
ADFI	Average daily feed intake
ADG	Average daily gain
F/G	Feed/gain ratio
ALT	Alanine amino transferase
AST	Aspartate amino transferase
ALP	Alkaline phosphatase
LDH	Lactate dehydrogenase
BUN	Blood urea nitrogen urea
UA	Uric acid
CHOL	Cholesterol
TG	Triglyceride
HDL	High-density lipoprotein
LDL	Low-density lipoprotein
GLU	Glucose
T-AOC	Total antioxidant capacity
MDA	Malondialdehyde
GSH-Px	Glutathione peroxidase
SOD	Superoxide dismutase
CAT	Catalase
VH	Villus height
CD	Crypt depth
MT	Muscularis thickness
VH/CD	Villus height-to-crypt depth ratio

## References

[1] Hou S.S., Liu L.Z. (2024). Report on the development of waterfowl industry and technology in 2023. Chin. J. Anim. Sci..

[2] Chen Z.M., Liu G.H. (2020). Research progress in development and utilization of poultry feed resources. Chin. J. Anim. Nutr..

[3] Huang Y., Ma J.L., Wang Q.G., Dong G.Z., Xie M., Hou S.S., Liu Z.L., Wang C. (2017). A substitution effect of green sweet sorghum stalks for diet on the growth performance, carcass yields and meat quality in geese. Acta Vet. Zootech. Sin..

[4] Zhong H., Liu Z., Liang M., Wang Q., Wang Y., Luo Y., Sun J., Zhang C., Li Q., Wang C. (2020). Effects of supplementing geese with green sweet sorghum stalks on microbiota in segments of the gastrointestinal tract. S. Afr. J. Anim. Sci..

[5] Wang M., Li S.M., Shen D., Han G.F., Li Y.S., Dou X.H., Li C.M. (2021). Effects of dietary different whole-plant corn silage levels on growth performance, slaughter performance, meat quality and serum biochemical parameters of geese. Acta Vet. Zootech. Sin..

[6] Li Y.P., Wang Z.Y., Yang H.M., Xu L., Xie Y.J., Jin S.L., Sheng D.F. (2017). Effects of dietary fiber on growth performance, slaughter performance, serum biochemical parameters, and nutrient utilization in geese. Poult. Sci..

[7] Zhang D., Wang N., Li C., Xie Q., Tang S. (2019). Sweet sorghum—A high efficient and quality forage crop. Biotechnol. Bull..

[8] Sun Z.Q., Luo Y.N., Xiong Y., Gao R., Wu Z., Yu Z. (2021). Evaluation of sweet sorghum as a high-quality forage in pasture-livestock industry development. Chin. J. Grassl..

[9] Li S.S. (2018). Evaluation of Nutritional Value of Sweet Sorghum and Application in Dairy Production. Master’s Thesis.

[10] Yu X. (2020). Effects of Sweet Sorghum Silage on the Performance, Blood Biochemical Indexes and Rumen Function of Dairy Cows. Master’s Thesis.

[11] Zhou L.P., Wang S.T. (2023). Effects of different levels of sweet sorghum in basic diet on growth performance, rumen fermentation, and slaughtering performance of fattening cattle. China Feed..

[12] Amar A. (2020). Effects of Sweet Sorghum and Alfalfa Mixed Storage on Growth Performance, Blood Biochemistry and Meat Quality of Karakul Sheep. Master’s Thesis.

[13] Wang J. (2021). Effects of Mixed Silage of Sweet Sorghum and Alfalfa on Tissue Morphology, Enzyme Activity and Flora of Karakul Sheep Digestive Tract. Master’s Thesis.

[14] Wang F., He Z.F., Chen P., Xie J.P., He C.G. (2023). Effect of sweet sorghum silage replacing maize silage on blood physiology, serum biochemical, and serum antioxidant indexes of sheep. Pratacultural Sci..

[15] Wang F., He Z.F., Chen P., He C.G. (2021). Effects of sweet sorghum hay replacement by different proportions of sweet sorghum silage on growth performance, nutrient digestibility and serum biochemical indexes of Dezhou donkey foals. Chin. J. Anim. Nutr..

[16] Chen X.W. (2024). Effects of Mixed Fermentation of Impurity from Machine-Picked Cotton and Sweet Sorghum on Serum Biochemical Indices, Intestinal Morphology and Microflora of Meat Rabbits. Master’s Thesis.

[17] Li L.L. (2024). Nutritional Characteristics of Machine-Harvested Cotton Residue and Sweet Sorghum Fermented Feed and Their Effects on Growth and Slaughter Performance of Meat Rabbit. Master’s Thesis.

[18] Zhan J.S., Xia C., Liu S.J., Zhou M.L., Yang H.B., Lin M., Liu M.M., Zhao G.Q. (2015). Effects of ryegrass on growth, carcass traits and blood biochemical indices of Yangzhou geese. Acta Prataculturae Sin..

[19] Zhan J.S., Zhan K., Huo Y.J., Lin M., Zhao G.Q., Yang F.Y. (2015). Effects of alfalfa pellet feed on growth performance, intestinal length and serum parameters of geese. J. China Agric. Univ..

[20] Zhan J.S., Zhan K., Liu M.M., Huo Y.J., Lin M., Zhao G.Q., Yang F.Y. (2015). Effects of alfalfa pellet feed on slaughter performance, organ weights and blood biochemical indices of geese. Acta Prataculturae Sin..

[21] Abdollahi M.R., Ravindran V., Svihus B. (2013). Pelleting of broiler diets: An overview with emphasis on pellet quality and nutritional value. Anim. Feed Sci. Technol..

[22] Goodarzi Boorejeni F., Svihus B., von Reichenbach H.G., Zentek J. (2016). The effects of hydrothermal processing on feed hygiene, nutrient availability, intestinal microbiota and morphology in poultry—A review. Anim. Feed Sci. Technol..

[23] Abdollahi M.R., Zaefarian F., Ravindran V., Selle P.H. (2018). The interactive influence of dietary nutrient density and feed form on the performance of broiler chickens. Anim. Feed Sci. Technol..

[24] National Research Council (1994). Nutrient Requirements of Poultry.

[25] Liu Z.L., Xue J.J., Huang X.F., Luo Y., Liang M.R., Li C.J., Wang Q.G., Wang C. (2020). Effect of feeding frequency on the growth performance, carcass traits, and apparent nutrient digestibility in geese. Poult. Sci..

[26] Liu Z.L., Xue J.J., Huang X.F., Chen Y., Wang Q.G., Zhang S., Wang C. (2021). Effect of stocking density on growth performance, feather quality, serum hormone, and intestinal development of geese from 1 to 14 days of age. Poult. Sci..

[27] Lv X., Chen L., Zhou C., Zhang G., Xie J., Kang J., Tan Z., Tang S., Kong Z., Liu Z. (2023). Application of different proportions of sweet sorghum silage as a substitute for corn silage in dairy cows. Food Sci Nutr..

[28] Li S.S., Zhang J.J., Bai Y.F., Allan Degen A., Wang T., Shang Z.H., Ding L.M., Long R.J. (2020). Sorghum silage substituted for corn silage in diets for dairy cows: Effects on feed intake, milk yield and quality, and serum metabolites. Appl. Anim. Sci..

[29] Ran T., Tang S.X., Yu X., Hou Z.P., Hou F.J., Beauchemin K.A., Yang W.Z., Wu D.Q. (2021). Diets varying in ratio of sweet sorghum silage to corn silage for lactating dairy cows: Feed intake, milk production, blood biochemistry, ruminal fermentation, and ruminal microbial community. J. Dairy Sci..

[30] Babu J., Kumari N.N., Reddy Y.R., Raghunandan T., Sridhar K. (2015). Effect of feeding sweet sorghum stover-based complete rations on the growth performance and carcass characteristics of ram lambs. Trop. Anim. Health Prod..

[31] Wu P., Fu X., Wang H., Hou M., Shang Z. (2021). Effect of silage diet (sweet sorghum vs. whole-crop corn) and breed on growth performance, carcass traits, and meat quality of lambs. Animals.

[32] Kokoszyński D., Bernacki Z., Grabowicz M., Stańczak K. (2014). Effect of corn silage and quantitative feed restriction on growth performance, body measurements, and carcass tissue composition in White Kołuda W31 geese. Poult. Sci..

[33] Jha R., Mishra P. (2021). Dietary fiber in poultry nutrition and their effects on nutrient utilization, performance, gut health, and on the environment: A review. J. Anim. Sci. Biotechnol..

[34] Wang X., Li G., Liu Y., Yang Y., Wang C., Gong S., Zhu L., Lei H., Wang H., He D. (2023). The effects of whole-plant silage maize as replacement commercial feed on the growth performance, carcass yield, relatively organ weight, blood biochemical, and economical traits in Holdobaki goose. Cogent Food Agric..

[35] Chen F., He J., Wang X., Lv T., Liu C., Liao L., Li Z., Zhou J., He B., Qiu H. (2022). Effect of dietary ramie powder at various levels on the growth performance, meat quality, serum biochemical indices and antioxidative capacity of yanling white geese. Animals.

[36] Valvona C.J., Fillmore H.L., Nunn P.B., Pilkington G.J. (2016). The regulation and function of lactate dehydrogenase a: Therapeutic potential in brain tumor. Brain Pathol..

[37] Rock K.L., Kataoka H., Lai J.J. (2013). Uric acid as a danger signal in gout and its comorbidities. Nat. Rev. Rheumatol..

[38] Yu J., Wang Z.Y., Yang H.M., Xu L., Wan X.L. (2019). Effects of cottonseed meal on growth performance, small intestinal morphology, digestive enzyme activities, and serum biochemical parameters of geese. Poult. Sci..

[39] Kurata M., Suzuki M., Agar N.S. (1993). Antioxidant systems and erythrocyte life-span in mammals. Comp. Biochem. Physiol. Part B Comp. Biochem..

[40] Castillo C., Hernández J., Valverde I., Pereira V., Sotillo J., López Alonso M., Benedito J.L. (2006). Plasma malonaldehyde (MDA) and total antioxidant status (TAS) during lactation in dairy cows. Res. Vet. Sci..

[41] Luo Q., Cui X., Yan J., Yang M., Liu J., Jiang Y., Li J., Zhou Y. (2011). Antagonistic effects of Lycium barbarum polysaccharides on the impaired reproductive system of male rats induced by local subchronic exposure to 60Co-γ irradiation. Phytother. Res..

[42] Khosravi M., Rouzbehan Y., Rezaei M., Rezaei J. (2018). Total replacement of corn silage with sorghum silage improves milk fatty acid profile and antioxidant capacity of Holstein dairy cows. J. Dairy Sci..

[43] Zhang Y.K., Xue J.J., Chen Y., Huang X.F., Liu Z.L., Zhong H., Xie Q., Luo Y., Wang Q.G., Wang C. (2024). Modulation of performance, plasma constituents, small intestinal morphology, and cecum microbiota in growing geese by dietary citric acid supplementation. Animals.

[44] Murakami A.E., Sakamoto M.I., Natali M.R.M., Souza L.M.G., Franco J.R.G. (2007). Supplementation of glutamine and vitamin E on the morphometry of the intestinal mucosa in broiler chickens. Poult. Sci..

[45] Laudadio V., Passantino L., Perillo A., Lopresti G., Passantino A., Khan R.U., Tufarelli V. (2012). Productive performance and histological features of intestinal mucosa of broiler chickens fed different dietary protein levels. Poult. Sci..

